# Nonlinear Modeling of Surface Roughness in Turning of Nitinol: Influence of Cutting Forces, Temperature, and Negative Rake Angle Tools

**DOI:** 10.3390/ma19183912

**Published:** 2026-09-15

**Authors:** Wojciech Zębala, Andrzej Matras, Mateusz Seltenreich

**Affiliations:** Department of Production Engineering, Faculty of Mechanical Engineering, Cracow University of Technology, 31-864 Kraków, Poland; wojciech.zebala@pk.edu.pl (W.Z.); m.seltenreich@doktorant.pk.edu.pl (M.S.)

**Keywords:** NiTi machining, nitinol turning, surface roughness, nonlinear regression, cutting forces, temperature, CBN tools, ceramic tools

## Abstract

**Highlights:**

- Nonlinear model of surface roughness including cutting forces and temperature.
- Feed rate identified as the dominant factor affecting Ra.
- Ceramic tools generate higher temperature and more regular surface topography.
- CBN tools ensure lower cutting forces and improved roughness parameters values.
- Strong thermo-mechanical coupling governs NiTi machining process.

**Abstract:**

This study investigates the influence of cutting parameters on surface roughness and process conditions during turning of NiTi (Nitinol), a difficult-to-machine shape memory alloy. Special attention was given to the coupling between mechanical and thermal phenomena governing surface formation. Experiments were conducted using cubic boron nitride (CBN) and ceramic tools with negative rake angle geometry. A two-factor design of experiments (DOE) was applied, with cutting speed (vc) and feed rate (f) as input variables, while cutting depth remained constant. Cutting force (Fc), temperature (T), and surface roughness parameters (Ra) were measured. Based on experimental data, a nonlinear regression model was developed. The model parameters were determined using the least squares method following a prior logarithmic transformation of the equation, which enabled the nonlinear regression problem to be reduced to a linear form. Results indicate that feed rate is the dominant factor affecting surface roughness, while cutting speed primarily influences temperature. Ceramic tools generate higher forces and temperatures but promote more regular surface geometry. In contrast, CBN tools provide lower forces and improved surface roughness, though with increased smearing effects. The study confirms that surface roughness in NiTi machining is governed by coupled thermo-mechanical interactions. The proposed model enables improved prediction and optimization of machining parameters, particularly for high-precision applications.

## 1. Introduction

Nickel-titanium (NiTi) alloys, commonly referred to as Nitinol, are widely used in advanced engineering due to their unique functional properties, such as the shape memory effect and superelasticity [1,2,3,4]. These characteristics enable their use in systems requiring large reversible deformations, corrosion resistance, and high reliability under cyclic loading [5,6,7].

The most demanding applications of NiTi alloys occur in the biomedical industry, where this material is used to manufacture vascular stents, guides, and surgical implants. In these applications, surface integrity plays a crucial role, directly affecting biocompatibility, corrosion resistance, fatigue life, and tribological properties during operation. Even small surface irregularities can act as stress concentrators, leading to reduced fatigue strength and the initiation of local corrosion processes [8,9,10,11]. Consequently, stringent surface quality requirements are imposed, including low roughness, absence of micro-damage, and controlled and predictable topography. Meeting these requirements is particularly difficult due to the poor machinability of NiTi alloys resulting from their strong tendency to strain hardening, high plasticity and low thermal conductivity [5,12,13,14,15].

In addition to biomedical applications, NiTi alloys are also finding increasing use in the aerospace and precision engineering industries, including adaptive structures, actuators, and micromechanical components. In these cases, the functional properties of the components depend not only on the bulk properties of the material but also on the condition of the surface layer. Surface defects can lead to reduced dimensional accuracy, instability under cyclic loading, and degradation of the shape memory effect. Therefore, strict control of surface integrity is also required in these applications [16,17,18,19,20].

These requirements impose significant constraints on machining processes. It has been shown that surface integrity in the machining of NiTi alloys is determined by coupled thermos-mechanical phenomena, including cutting forces, temperature, and phase transformations [18,19,20,21,22]. Asadi et al. emphasized that the thermomechanical state of NiTi alloys strongly de-pends on the phase equilibrium of martensite and austenite. Even small temperature changes can alter the functional properties of the material, confirming the importance of physical phenomena occurring during machining processes [23]. Additionally, Wang et al. showed that feed-related machining parameters significantly influence the surface profile, strain hardening behavior, and resulting surface morphology of NiTi shape memory alloys, which highlights the importance of considering thermo-mechanical phenomena during process optimization [24]. Due to the material’s low thermal conductivity, a significant portion of the generated heat accumulates in the cutting zone, leading to thermal plasticization, phase transformations between austenite and martensite, and intensified adhesion and tool wear. These phenomena have a direct impact on both the surface formation mechanism and the final quality of the surface layer [13,22,25,26].

In this context, surface roughness cannot be considered solely as a geometric effect of the cutting process, but should be treated as the direct outcome of coupled thermal and mechanical interactions occurring within the machining zone. Under these conditions, surface layer generation is strongly governed by the complex interplay between process energy, localized heat generation, phase transformations, and material behavior during severe plastic deformation.

General recommendations for machining NiTi alloys typically recommend a positive rake angle (γ > 0), as this reduces cutting forces, limits strain hardening, and improves surface quality [13,27,28,29].

NiTi alloys are characterized by specific technological properties, including high elasticity (superelasticity), strong adhesion to the cutting edge, and a tendency for the material to flow plastically rather than shear cleanly. The use of a positive rake angle gives the cutting edge an “aggressive” character, which, in the case of nitinol, can lead to undesirable material stretching, sticking to the rake face, and the initiation of built-up edges and smearing [12,30,31,32].

The use of a negative rake angle (γ < 0) significantly increases cutting edge stability and reduces the occurrence of adhesion phenomena typical of NiTi alloys. Negative geometry translates into higher mechanical strength of the cutting edge, which is crucial for tools made of brittle materials such as ceramics or CBN (cubic boron nitride). This effectively minimizes the risk of chipping and micro-cracks.

Despite the general recommendation to use positive rake angles in machining difficult-to-cut materials, CBN and black ceramic tools with negative rake angles were used in this study.

Analyzing the cutting mechanism, it should be noted that a negative rake angle generates higher compressive forces in the area in front of the cutting edge, which forces more controlled shearing of the material, reducing the “pulling” and surface smearing phenomena characteristic of positive geometry tools [33]. Furthermore, due to increased unit pressures, the formation of a stable built-up edge is hindered—the material is more effectively “pushed” from the machining zone rather than being plastically stretched on the machined surface. Furthermore, a negative rake angle induces an increase in temperature in the cutting zone, which, when using ceramic tools, stabilizes the process by thermally plasticizing the material.

Despite extensive research on NiTi machining, most existing empirical models predict surface roughness solely as a function of kinematic parameters, neglecting the strong thermo-mechanical coupling inherent to shape memory alloys. Furthermore, while positive rake geometries are conventionally recommended, their tendency to cause severe material pulling and adhesion remains a challenge, leaving the potential of negative rake geometries using hard tool materials (CBN and ceramic) insufficiently explored. To address this research gap, this study aims to investigate the coupled thermo-mechanical interactions during the turning of Nitinol with negative rake angle CBN and ceramic tools. The primary objective is to develop an extended nonlinear empirical surface roughness model that explicitly incorporates cutting force (F_c_) and cutting temperature (T).

The main innovation of this work is to identify the trade-offs between ceramic and CBN tools in negative rake geometries and to demonstrate how physical process parameters can be integrated into a highly accurate nonlinear model for predicting surface roughness in difficult-to-cut NiTi alloys. Although incorporating cutting force (F_c_) and temperature (T) requires additional instrumentation, this hybrid approach offers distinct statistical and physical advantages over traditional parametric models. First, Nitinol exhibits non-linear thermo-mechanical behaviors—such as stress-induced phase transformation and severe work hardening—which cannot be captured by kinematic cutting parameters (v_c_, f) alone. F_c_ and T act as real-time physical state signatures reflecting tool wear, elastic recovery, and localized thermal softening. Consequently, this model achieves superior predictive robustness and provides a theoretical foundation for in-situ surface quality monitoring in advanced manufacturing environments.

## 2. Materials and Methods

### 2.1. Characteristics of the Workpiece and Cutting Tools

The material used in the study was a 50 mm diameter shaft made of a nickel-titanium alloy (NiTi) with a chemical composition of 50% Ni and 50% Ti. The alloy’s properties are presented in Table 1.

Two types of indexable cutting inserts from Seco Tools were used in the study. The first tool was the CNGN120708S-01020 CS100 ceramic insert (SECO Tools AB, Fagersta, Sweden) from the Secomax series. The CS100 grade was developed for machining materials that generate high thermal loads, such as titanium alloys and heat-resistant superalloys. Thanks to their high resistance to abrasive wear and the retention of cutting properties at elevated temperatures, ceramic inserts enable higher cutting speeds than tools made of cemented carbide. The second tool used was the CNGA120408E25-L1-U CBN170 insert (SECO Tools AB, Fagersta, Sweden), made of polycrystalline boron nitride (CBN). This material is among the hardest tool materials used in machining and is characterized by very high wear resistance, thermal stability, and the ability to maintain a sharp cutting edge. Thanks to these properties, CBN tools are suitable for machining difficult-to-cut materials and materials requiring high dimensional accuracy and surface quality. Detailed geometric and design parameters of both inserts are presented in Table 2.

The cutting inserts were mounted in a DCLNR 2020K12 holder (Sandvik Coromant, Sandviken, Sweden) designed for external turning. It features a 20 × 20 mm shank cross-section, 125 mm length, right-hand design, a 95° lead angle, and a 6° clearance angle. The holder geometry corresponds to a negative rake angle of −6°.

### 2.2. Measurement Equipment Used

A FLIR SC 620 thermal imaging camera (Teledyne FLIR, Wilsonville, OR, USA), equipped with a 38 mm fixed lens, was used to monitor and analyze the temperature distribution in the machining area. Thermal images were captured and analyzed using FLIR Thermal Studio software version 2.0.58.0. Thermograms were recorded at a resolution of 640 × 480 pixels at an acquisition frequency of 30 Hz. During the tests, the material’s emissivity value, determined experimentally, was assumed to be 0.90. The camera was positioned approximately 1 m from the test sample, and the temperature measurement range was 0 to 500 °C.

**Figure 1:**
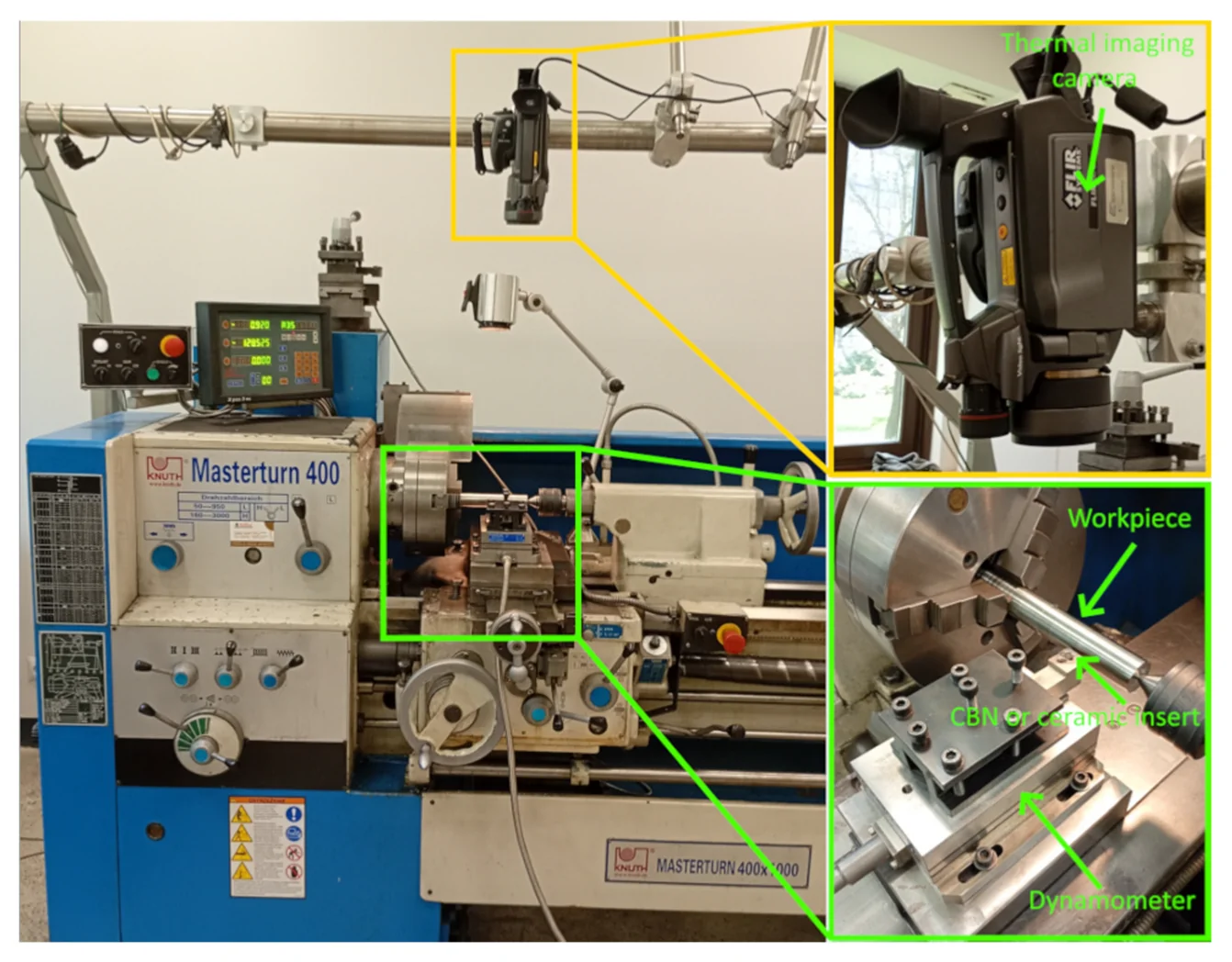
Experimental stand used during NiTi turning experiments.

The total cutting force components were measured using a Kistler 9257B piezoelectric dynamometer (Kistler, Winterthur, Switzerland). Signals from the dynamometer were transmitted to a Kistler 5070B charge amplifier (Kistler, Winterthur, Switzerland), and the results were recorded and analyzed using the Kistler DynoWare 2825A software (Kistler, Winterthur, Switzerland). The experimental stand used for the turning tests is shown in Figure 1.

Surface roughness parameters were determined using a Talysurf Intra 50 profilometer (Taylor Hobson, Leicester, UK). Measurements were taken along the tool feed direction using a measuring tip with a radius of 2 μm. Roughness parameter evaluation was performed in accordance with the requirements of the ISO 4287 standard [34]. The cutoff wavelengths used during the measurements were λc = 0.8 mm and λs = 2.5 μm. Each measurement was performed over a 5 mm long section.

Microscopic analysis of the surface was performed using a VHX-600 digital optical microscope (Keyence, Osaka, Japan) operating at 200× magnification.

### 2.3. Experimental Design

The experimental design used corresponds to a full two-factor experimental design, in which the input variables were cutting speed v_c_ and feed f. The parameters analyzed included changes within the following ranges:

- Cutting speed (v_c_): 40; 50; 60 m/min
- Feed per revolution (f): 0.09; 0.125; 0.16 mm/rev

The depth of cut was kept constant at a_p_ = 0.5 mm, representing typical finish turning conditions. In finish machining, ap has a minor influence on surface roughness parameters compared to feed rate and cutting speed, while a constant a_p_ prevents force fluctuations that could induce dynamic chatter in low-rigidity Nitinol workpieces.

Tests were performed in a random order. Measurements were repeated three times, allowing for the assessment of process repeatability and the identification of machining instability, particularly significant in the case of NiTi alloys. This is crucial for the analysis of cutting forces, temperature analysis in the cutting zone, and the identification of nonlinear regression models. The research design was developed to identify a nonlinear surface roughness model that takes into account mechanical and thermal phenomena in the cutting process.

(1) $$Ra=C\cdot v_{c}^{a}\cdot f^{b}\cdot F_{c}^{d}\cdot e^{\gamma T}$$

where:

*Ra*: arithmetic mean deviation of the roughness profile,

*C*: material-process constant,

*v_c_*: cutting speed,

*f:* feed,

*F_c_:* cutting force,

$e^{\gamma T}$: exponential term representing the effect of temperature (T) in the cutting zone, where *γ* is the correlation coefficient.

The proposed equation is not a classical model for predicting surface roughness based on cutting parameters, but a thermo-mechanical model describing the mechanisms of surface formation during NiTi turning. Although cutting force (F_c_) and temperature (T) are dependent on process parameters, they are also physical quantities that directly characterize the actual state of the cutting zone. In the case of NiTi alloys, the process is strongly dependent on local temperature, plastic deformation, phase transformations, elastic material recovery, and adhesion phenomena. These phenomena cannot be fully described by cutting speed and feed rate alone. Therefore, including cutting force and temperature in the model allows for a better representation of the actual thermos-mechanical conditions of the process and the mechanisms responsible for surface roughness formation.

## 3. Research Results and Analysis

Table 3 presents the results of the NiTi (nitinol) alloy turning tests for two tools—CBN and Ceramic.

The following chapters present some important conclusions regarding cutting forces, temperature and machined surface quality.

### 3.1. Cutting Forces (Fc)

For cubic boron nitride (CBN) tools, average cutting force values were recorded in the range of 247–393 N. The lowest values were recorded for the parameters v_c_ = 50 m/min and f = 0.09 mm/rev, where the cutting force was approximately 247 N. The increase in F_c_ was observed primarily with increasing feed, while the effect of cutting speed was moderate. For both tool materials and feed f = 0.09 mm/rev, the cutting force decreases as cutting speed increases from 40 to 50 m/min, indicating the dominance of thermal softening. When the cutting speed reaches 60 m/min, the cutting force increases due to intensified friction, adhesion, and increased contact stress at the tool-workpiece interface. Similar trends were observed for f = 0.16 mm/rev. When using a feed rate f = 0.125 mm/rev and a ceramic tool, a different trend is observed. The cutting force increases from 301 N to 321 N when the cutting speed rises from 40 to 50 m/min, suggesting that undesirable thermo-mechanical phenomenon dominates over thermal softening. At the highest cutting speed (60 m/min), the increased temperature theoretically promotes material softening, leading to a reduction in the cutting force to 314 N. For the CBN tool, only minor changes in Fc were observed (281–289 N), indicating a balance between thermal softening and competing thermo-mechanical phenomena. These results demonstrate that the machining response of NiTi alloys is determined by a complex interaction between thermal softening, thermo-mechanical effects, and adhesion phenomena, resulting in a nonlinear variation of the cutting force.

**Figure 2:**
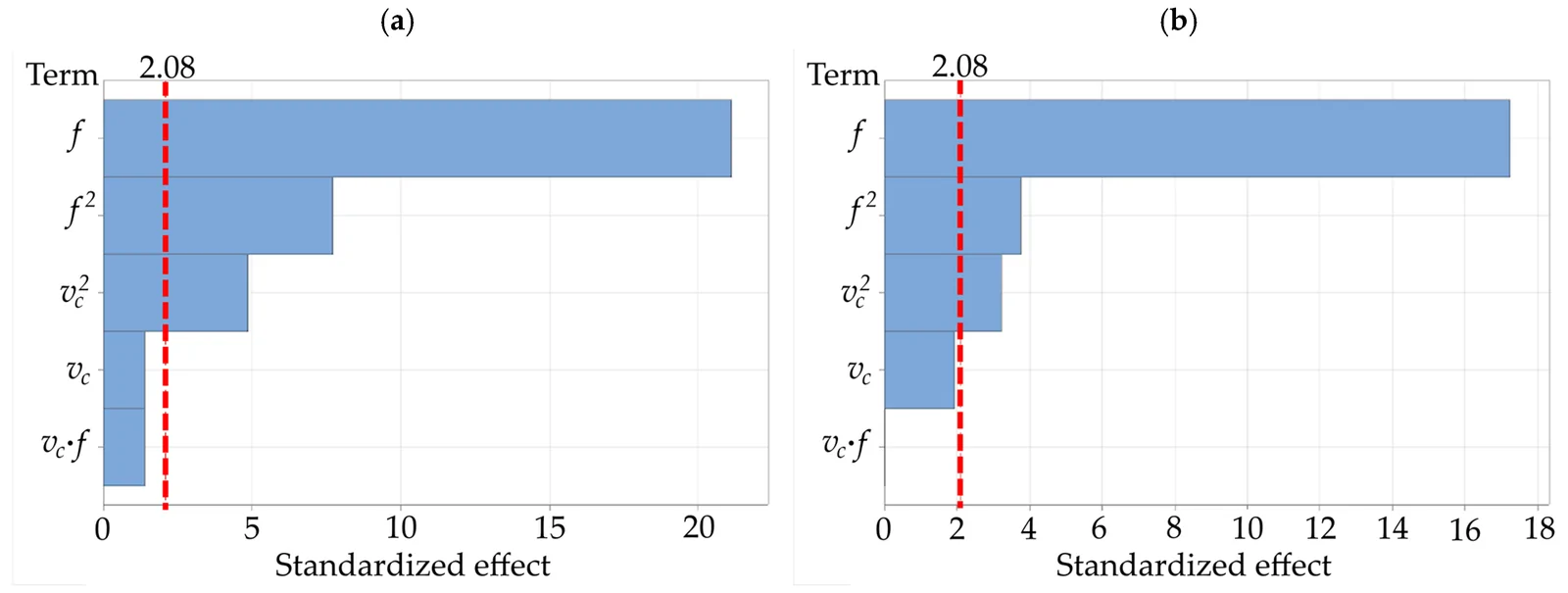
Pareto analysis of the effect of feed and cutting speed on cutting force values: (**a**) CBN tool; (**b**) Ceramic tool.

Pareto diagrams (for the assumed α = 0.05) performed to assess the significance of the effect of the tested cutting parameters on the cutting force values are shown in Figure 2a,b.

**Figure 3:**
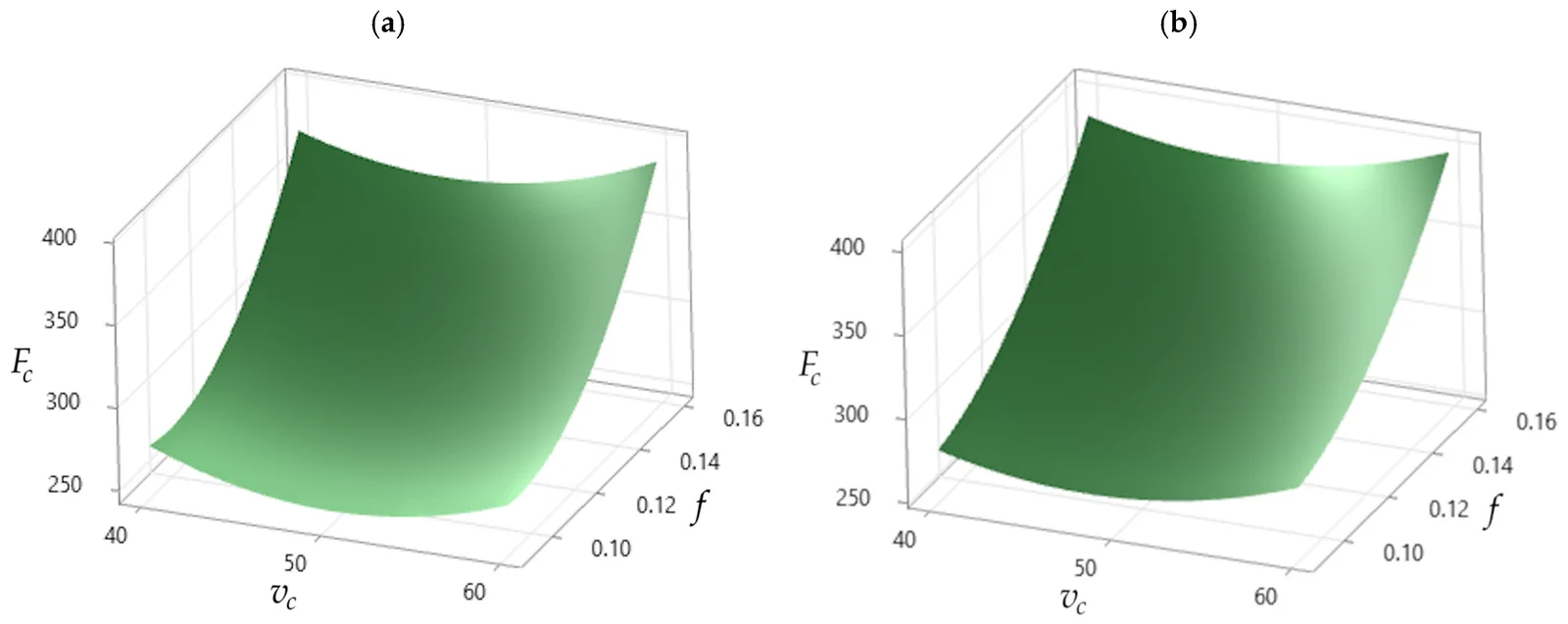
Effect of feed rate and cutting speed on cutting force values: (**a**) CBN tool; (**b**) Ceramic tool.

For ceramic tools, the cutting force range was higher, ranging from 259 to 405 N. Figure 3a,b show the effect of cutting speed and feed on cutting force values.

The cutting force F_c_ can be decomposed into components corresponding to the shearing mechanisms F_shear_, friction F_friction_ and compression F_compression_ (2):

(2) $$F_{c}=F_{shear}+F_{friction}+F_{compression}$$

Negative rake angles increase the friction and compression components, leading to an increase in the total cutting force and a shift in the nature of the process toward a predominance of contact interactions. The use of CBN tools results in lower Fc values, which can be attributed to the high thermal conductivity of this material and the reduction of local overheating in the cutting zone. Ceramic tools, on the other hand, generate higher cutting forces, accompanied by an increase in temperature in the machining zone.

### 3.2. Temperature in the Cutting Zone

For CBN tools, the temperature in the cutting zone ranged from 330 to 383 °C, demonstrating a relatively stable course. This can be attributed to the high thermal conductivity of CBN, which promotes more efficient heat dissipation from the cutting zone. The observed temperature variations result from the combined influence of cutting speed, feed rate, and the changing balance between plastic deformation and frictional phenomena occurring at the tool-workpiece interface.

For ceramic tools, significantly higher temperatures were observed, ranging from 363 to 463 °C, with the highest values occurring at v_c_ = 60 m/min. The lower thermal conductivity of ceramic tool limits heat transfer away from the cutting zone, leading to heat accumulation at the tool-workpiece interface. The increase in cutting speed intensified heat generation, while the simultaneous occurrence of thermal softening and adhesion phenomena contributed to the nonlinear temperature response.

**Figure 4:**
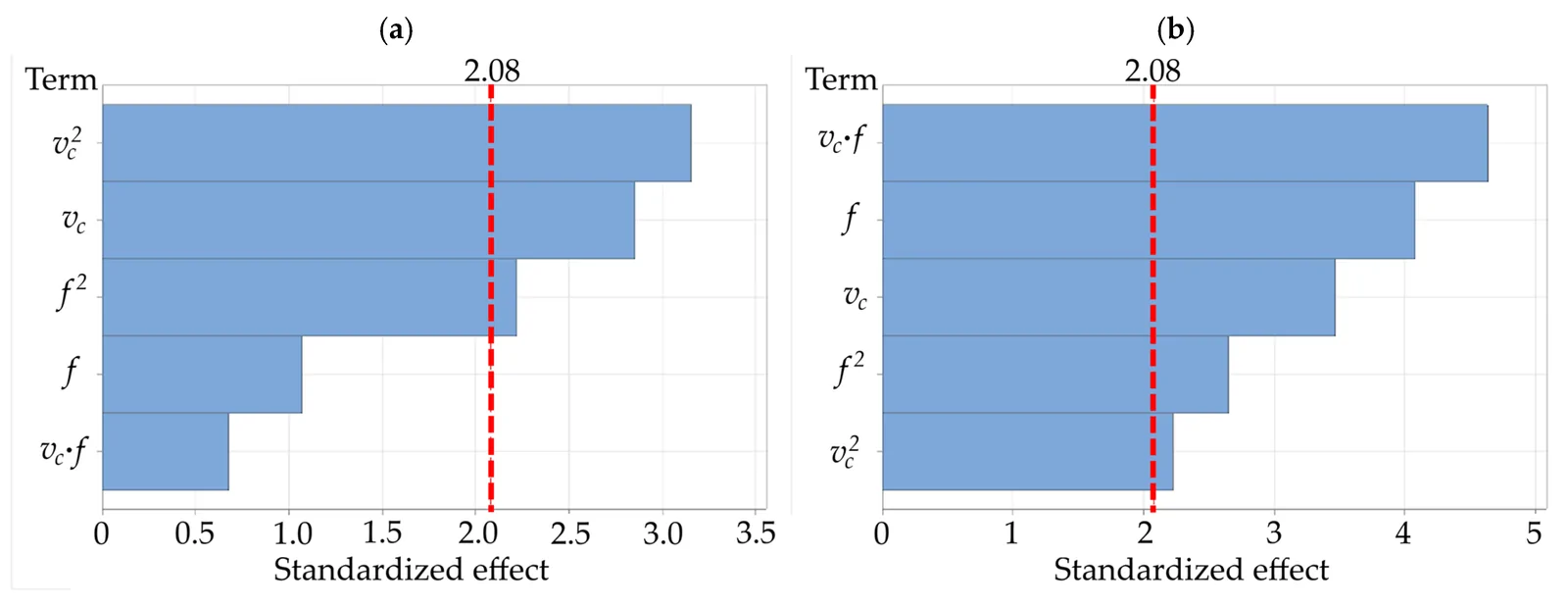
Pareto analysis of the effect of feed and cutting speed on the temperature values: (**a**) CBN tool; (**b**) Ceramic tool.

Pareto diagrams (assuming α = 0.05) created to assess the significance of the effect of the tested cutting parameters on the temperature values in the cutting zone are shown in Figure 4a,b.

**Figure 5:**
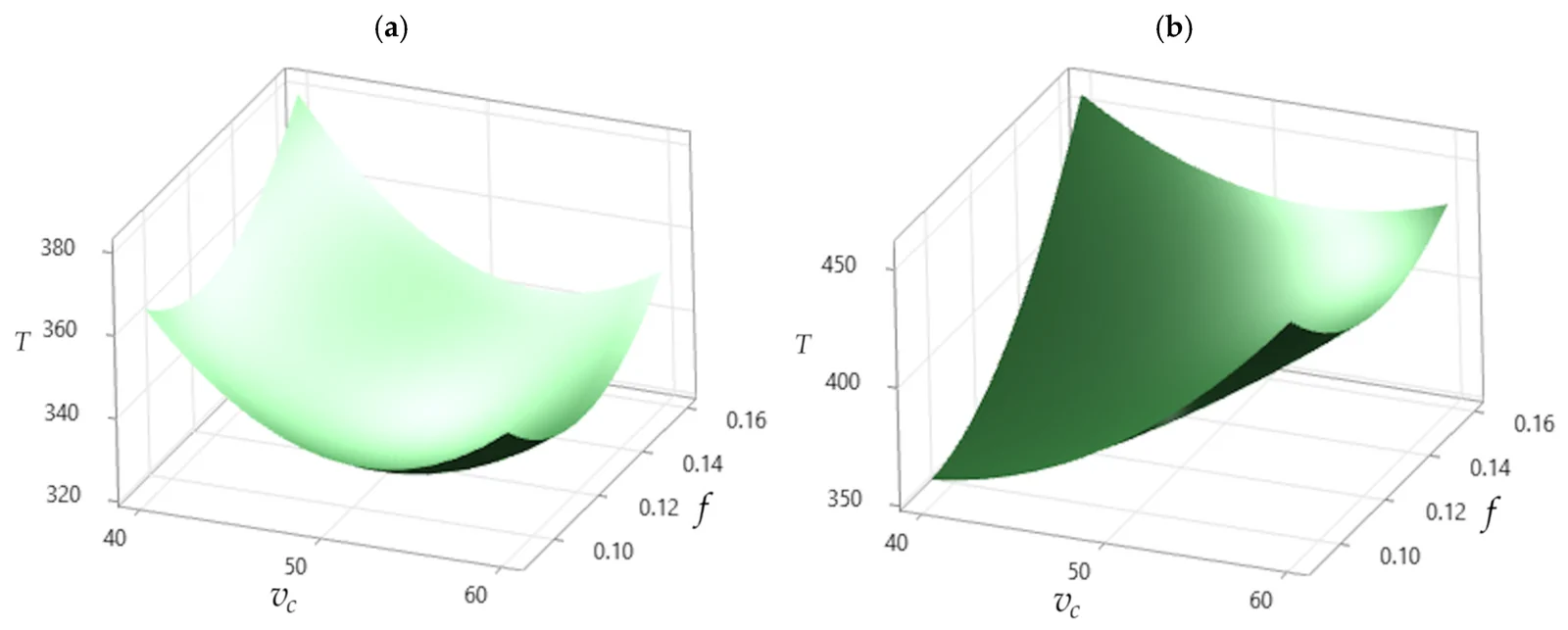
Effect of feed rate and cutting speed on machining temperature values: (**a**) CBN tool; (**b**) Ceramic tool.

In both cases, a nonlinear effect of cutting speed and feed rate was observed. Figure 5a,b show the effect of cutting speed and feed rate on temperature values in the cutting zone.

### 3.3. Effect of Cutting Parameters on Surface Roughness

For CBN tools, roughness values Ra ranged from 0.87 to 2.41 μm. The most favorable results were achieved for the parameters v_c_ = 50 m/min and f = 0.09 mm/rev, where Ra ≈ 0.87 μm. The results were characterized by relatively high repeatability. For ceramic tools, the Ra value range was 0.91 to 2.43 μm. The most favorable results were also achieved for the parameters v_c_ = 50 m/min and f = 0.09 mm/rev. Both tool types allow for similar roughness values.

Feed rate (f) is the dominant factor influencing the process. An increase in f causes a significant increase in cutting force Fc and simultaneously leads to a deterioration in surface roughness. Cutting speed primarily affects thermal phenomena:

Increasing the speed from 40 to 50 m/min causes a decrease in Ra by approximately:

- by 38% for f = 0.09,
- by 18% for f = 0.125,
- by 5% for f = 0.16.

This effect can be attributed to moderate thermal softening of the workpiece material, which improves chip formation and reduces mechanical interaction between the tool and the machined surface. With a further increase in cutting speed to 60 m/min, excessive heating and deterioration of surface roughness were observed, particularly for ceramic tools. Under these conditions, the higher temperature promotes adhesion phenomena and material smearing on the machined surface.

The obtained results indicate a strong coupling between the mechanical and thermal parameters of the process. The feed rate determines the intensity of deformation and the generated forces, while the cutting speed controls the thermal conditions, which are crucial in the case of NiTi alloys due to the potential for initiating phase transformations.

### 3.4. Surface Shaping Mechanisms

Significant differences in the surface generation mechanisms were observed depending on the tool material used. Ceramic tools generate higher cutting forces (approximately 20 N on average), leading to more intense plastic deformation in the shear zone. Under these conditions, the material is effectively cut off rather than deformed and dragged across the surface. The stable shearing mechanism and elevated temperature promote process stability and limit the formation of a built-up edge (BUE), which tends to be unstable under these conditions. This promotes the formation of a regular, periodic topographic structure consistent with the process kinematics, despite higher Ra values.

In contrast, surfaces machined with CBN tools are characterized by lower cutting forces, which limits global deformation of the workpiece. Lower process temperatures (approximately 60 °C on average) do not ensure complete thermal softening of the material. Instead of clean shearing, localized material drag is observed, resulting from:

- Microadhesion: thin layers of the machined alloy become interlocked with the cutting edge and are then stretched across the machined surface, creating a smearing effect.
- Spring-back effect: the significant elasticity of nitinol causes partial material recovery after the cutting edge passes, leading to the theoretical geometric profile being erased.
- Incomplete material separation: instead of forming a clean chip, some of the material is abraded and plastically rubbed onto the surface.

The final result is a surface machined with CBN tools characterized by more favorable (lower) Ra roughness parameters in quantitative terms, but its topography is irregular and blurred. Table 4 summarizes the key differences in geometry and physical mechanisms for both tool types.

### 3.5. Microscopic Analysis

**Figure 6:**
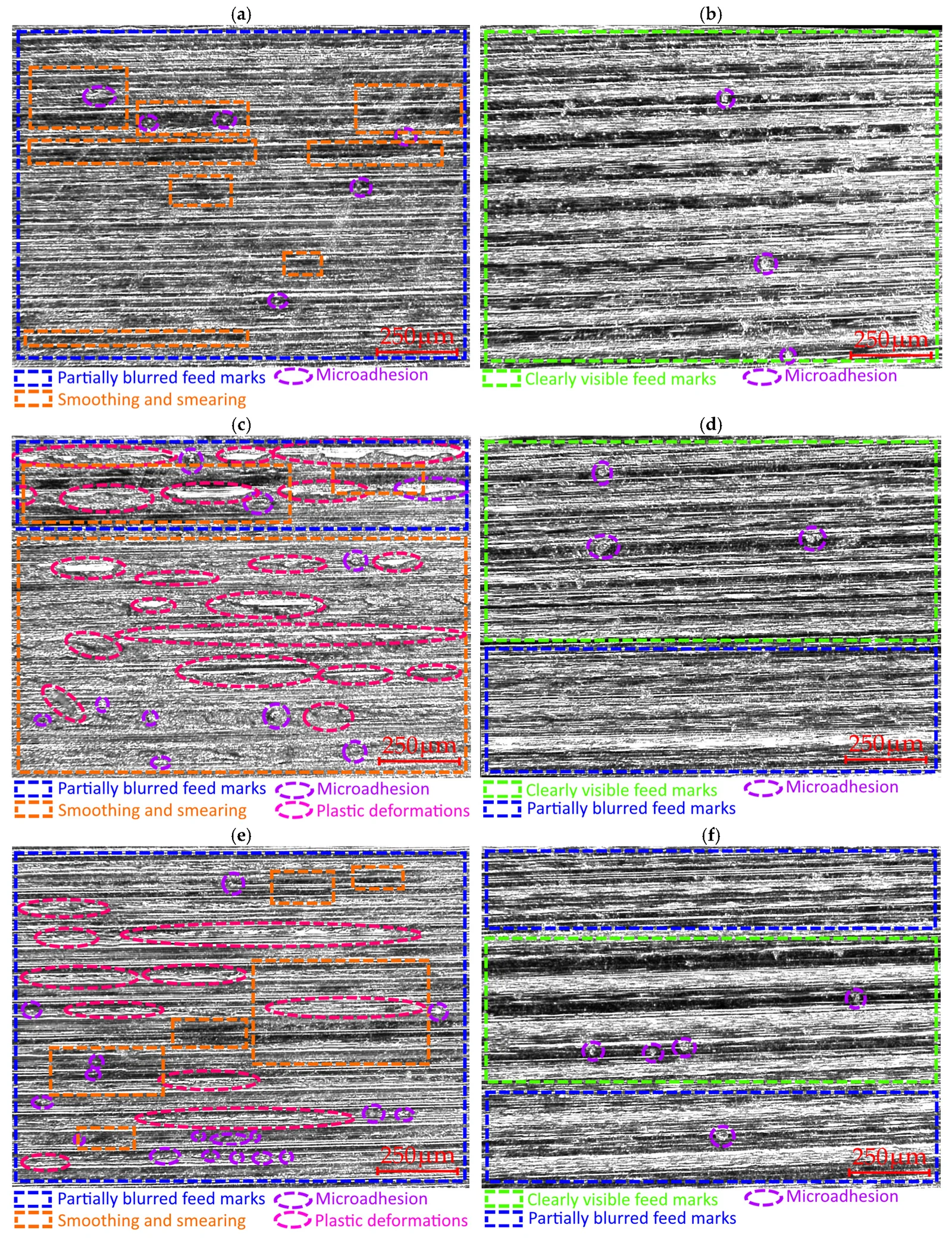
Microscopic images of the machined surfaces: v_c_ = 60 m/min: (**a**) f = 0.09 mm, CBN tool; (**b**) f = 0.09 mm, Ceramic tool; (**c**) f = 0.125 mm, CBN tool; (**d**) f = 0.125 mm, Ceramic tool; (**e**) f = 0.16 mm, CBN tool; (**f**) f = 0.16 mm, Ceramic tool.

Figure 6a–f present microscopic images of the machined surfaces, showing the characteristic turning marks produced at a cutting speed of 60 m/min and various feed rates.

Micrograph analysis of the surface after turning confirms differences in the mechanisms of surface layer formation between ceramic and CBN tools. For samples machined with a ceramic tool (Figure 6b,d,f), clearly defined, parallel feed marks are visible, corresponding to the kinematics of the turning process. The surface structure is regular and periodic, and the grooves maintain a relatively constant width and direction. This indicates the dominance of the material shearing mechanism and the preservation of the geometric representation of the tool movement on the machined surface.

However, for surfaces machined with CBN tools (Figure 6a,c,e), partial blurring and blurring of the machining marks are observed. In particular, Figure 6c shows local areas of smoothing and smearing of the material, which may be related to the occurrence of adhesion phenomena and the elastic-plastic nature of nitinol deformation. The boundaries between successive feed marks are less distinct than for ceramics, indicating partial abrasion of the material on the surface after the cutting edge passes. As the feed rate increases, the depth and spacing of machining marks increase for both tool materials. However, for ceramic tools, these marks remain legible and well defined, while for CBN tools, the proportion of smeared areas increases. This confirms that the surface after machining with ceramics is primarily geometric, resulting from a regular shearing process, while the surface after machining with CBN is shaped to a greater extent by physical-adhesive phenomena, such as microadhesion, local material drag, and springback. Table 5 summarizes the key features of the obtained surfaces.

## 4. Regression Model for the Surface Roughness Parameter Ra

To determine the exponents (a, b, d) and coefficient g in the Ra regression model (Equation (3)), the nonlinear model was transformed into a linear form using a natural logarithmic transformation:

(3) $$\ln\left(Ra\right)=\ln\left(C\right)+a\cdot \ln\left(v_{c}\right)+b\cdot \ln\left(f\right)+d\cdot \ln\left(F_{c}\right)+\gamma T$$

The temperature term was not logarithmically transformed because: $\ln\left(e^{\gamma \;T}\right)=\gamma \;T$.

After entering the markings:

- $Y=\ln\left(Ra\right)$
- $X_{1}=\ln\left(v_{c}\right)$
- $X_{2}=\ln\left(f\right)$
- $X_{3}=\ln\left(F_{c}\right)$
- $X_{4}=T$

which yields the following linear regression model:

(4) $$Y=\beta_{0}+aX_{1}+bX_{2}+dX_{3}+\gamma X_{4}$$

where: $\beta_{0}=ln(C)$.

The least squares method (LSM) was used to estimate the model coefficients. The following system of matrix equations is solved:

(5) $$\beta ={\left(X^{T}X\right)}^{-1}X^{T}\cdot Y$$

where:

$$\beta =\left[\begin{array}{c} ln(C)\\ a\\ b\\ d\\ \gamma \end{array}\right]$$

$$X=\left[\begin{array}{ccccc} 1 & \ln\left(v_{c}\right) & \ln\left(f\right) & \ln\left(F_{c}\right) & T\\ \vdots & \vdots & \vdots & \vdots & \vdots \end{array}\right]$$

and vector

$$Y=\left[\begin{array}{c} \ln\left(Ra\right)\\ \vdots \end{array}\right]$$

The LSM finds coefficients that minimize the sum of squared errors:

(6) $$S=\sum {\left(Y_{exp}-Y_{model}\right)}^{2}$$

(7) $$error=\ln\left(Ra_{exp}\right)-\ln\left(Ra_{model}\right)$$

**Figure 7:**
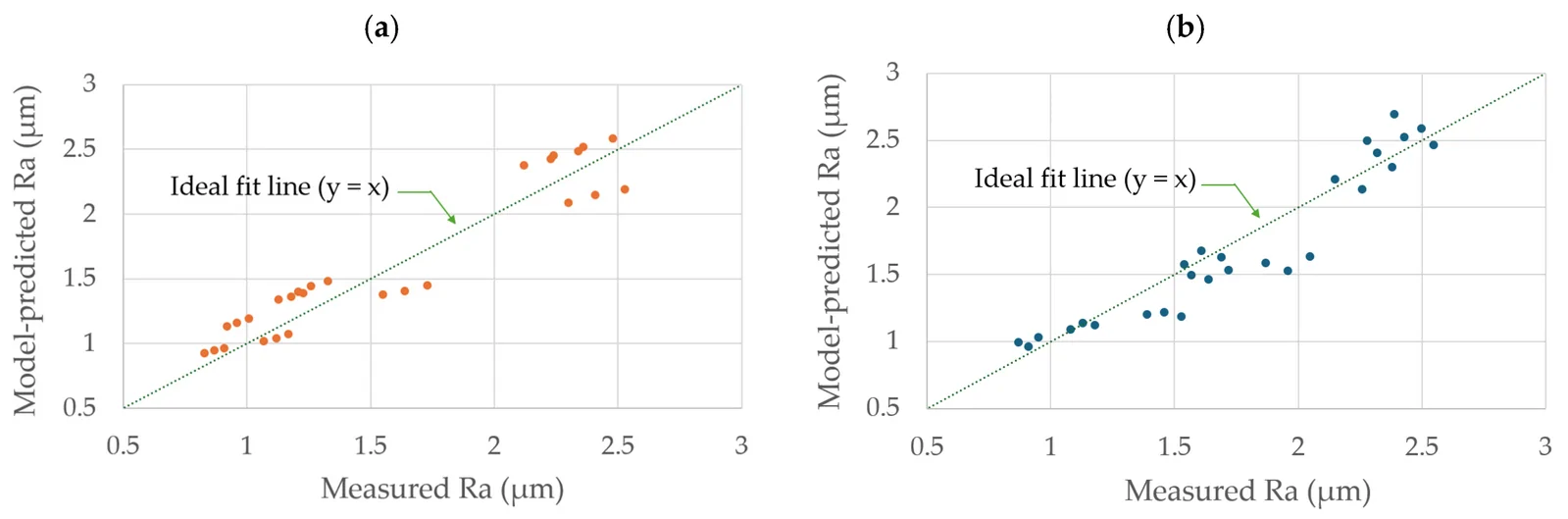
Measured versus predicted values of the surface roughness parameter Ra: (**a**) CBN Tool; (**b**) Ceramic Tool.

Regression analysis fits a hyperplane in the multidimensional space: (ln(*v_c_*), ln(*f*), ln(*F_c_*), *T*) to best predict ln(Ra) values. Model parameters were determined using the least squares method by minimizing the sum of squared deviations between the experimental values and the values calculated from the model. The obtained regression statistics are presented in Table 6. The presented regression statistics were determined based on a single experimental dataset, in which the results obtained for CBN and ceramic tools were analyzed simultaneously. Sets of data measured during the experiment and calculated using the model were compared. The comparison of the measured and predicted values of the Ra parameter is shown in Figure 7a,b.

The obtained multiple correlation coefficient value of R = 0.96 indicates a very strong correlation between the values predicted by the model and the experimental results. The coefficient of determination R^2^ = 0.91 indicates that the model explains approximately 91% of the variance in the surface roughness parameter Ra. The adjusted coefficient of determination, taking into account the number of explanatory variables, reached a value of 0.91, confirming the high quality of the fit and the absence of excessive model overfitting. The standard error of 0.174 indicates small deviations between the calculated and experimental values.

To assess the statistical significance of the model, an analysis of variance (ANOVA) was performed, the results of which are summarized in Table 7.

The very high F statistic value of 128.95 with a *p* value of <0.0001 indicates statistical significance for the entire regression model. This means that the included process variables significantly influence the surface roughness parameter, and the probability of obtaining such a fit as a result of random data fluctuations is negligible. Next, the coefficients of the regression equation were determined. After substituting the obtained coefficient values, the final model was obtained, described by Equation (8).

(8) $$Ra=0.0037284\cdot v_{c}^{-0.2586}\cdot f^{0.5584}\cdot F_{c}^{1.465}\cdot e^{-0.0005431T}$$

The validity of the developed model was verified by analyzing residuals and comparing calculated values with experimental results. A graph depicting the relationship between observed and model predicted values is presented in Figure 7a,b. The “Measured vs. Predicted” plot compares experimentally measured values with those predicted by the developed model. The distribution of points near the ideal fit line (x-y) suggests that the model describes the relationship between the measured and calculated values with satisfactory accuracy and without significant systematic errors.

## 5. Conclusions

The developed regression model accurately reproduces the experimental data, achieving a coefficient of determination of R^2^ = 0.91 and a mean relative error of MAPE = 9.1%. The analysis showed that, among the parameters studied, feed rate has the greatest impact on surface roughness. Process temperature and cutting force also exert a significant influence, although their interaction is coupled and depends on the material deformation conditions in the cutting zone. Cutting force acts as an energy coupling.

Analysis of the surface morphology revealed fundamental differences in the mechanisms of surface layer formation for both tool materials. In the case of CBN tools, the machining process is associated with a greater proportion of elastic-plastic deformations and adhesion phenomena, leading to the formation of surfaces with a more diffuse and heterogeneous topography. However, machining with ceramic tools promotes a stable material shearing mechanism, resulting in a more regular and periodic surface structure. An interesting phenomenon was observed: surfaces machined with CBN tools exhibit lower Ra values and less topographic regularity than surfaces obtained with ceramic tools. These results indicate that assessing surface layer quality based solely on the Ra parameter may be insufficient, especially in the case of NiTi alloys.

These results confirm the need for a combined analysis of roughness parameters, cutting forces, process temperature, and surface morphology. Tool material selection is a compromise between minimizing cutting forces, controlling thermal conditions, and the requirements for the surface layer structure.

These results may find practical applications in optimizing NiTi alloy machining processes, particularly in the selection of tool material and cutting parameters for components with high quality requirements.

This study also suggests several directions for future research. First, a detailed analysis of tool wear mechanisms should be conducted for both CBN and ceramic tools. Initial observations suggest that CBN inserts may exhibit higher wear rates than ceramic tools under the machining conditions studied. However, a comprehensive assessment of wear mechanisms, including flank wear, adhesion, micro-chipping, and their effects on cutting forces, temperature, and surface integrity, requires specialized studies.

Another research direction concerns the extension of the experimental design by including depth of cut as an additional input parameter. In the present study, the depth of cut was kept constant to enable a focused analysis of the thermo-mechanical effects associated with cutting speed and feed rate. However, increasing the cross-sectional area of the removed material may significantly affect cutting forces, heat generation, tool wear, and surface quality. Consequently, future studies should consider a three-factor experimental design incorporating cutting speed, feed rate, and depth of cut.

Furthermore, the observed nonlinear relationships between cutting parameters, cutting force, temperature, and surface roughness suggest that advanced modelling approaches based on machine learning or hybrid thermo-mechanical models may provide a more comprehensive description of the machining behavior of NiTi alloys.

## Figures and Tables

**Table 1:** Selected properties of NiTi alloy for martensitic and austenitic phases.

Property	Martensite	Austenite
Chemical composition (%)	50 Ni—50 Ti	
Density ρ (g/cm^3^)	6.45	
Melting temperature (°C)	1240–1310	
Young’s modulus E (GPa)	30–40	60–80
Tensile strength Rm (MPa)	800–1100	900–1500
Yield strength Rp0.2 (MPa)	200–400	500–700
Elongation A (%)	20–30	10–20
Hardness (HV)	200–250	250–350
Poisson’s ratio ν (-)	0.33	0.30
Thermal conductivity (W/(m·K))	8.6–12	12–18
Specific heat cp (J/(kg·K))	460–520	320–460

**Table 2:** Cutting insert parameters.

Parameter	CBN Tool	Ceramic Tool
Tool designation	CNGN120708S-01020	CNGA120408E25-L1-U
Grade	CS100	CBN170
Corner Radius (mm)	0.8	
Inscribed Circle Diameter (mm)	12.7	
Thickness (mm)	4.76	
Clearance angle (°)	0	
Number of cutting edges (-)	4	

**Table 3:** Cutting parameters and results obtained for CBN and ceramic tools.

	Cutting Parametr		CBN Tool			Ceramic Tool		
Case No.	$v_{c}$ (m/min)	f (mm/rev)	$F_{c}$ (N)	T (°C)	Ra (µm)	$F_{c}$ (N)	T (°C)	Ra (µm)
1	40	0.09	276	368	0.96	283	363	1.46
2	40	0.125	281	352	1.27	301	374	1.96
3	40	0.16	378	383	2.36	393	463	2.39
4	50	0.09	247	330	0.87	259	377	0.91
5	50	0.125	287	336	1.64	321	403	1.61
6	50	0.16	348	339	2.41	362	414	2.26
7	60	0.09	275	352	1.12	294	454	1.13
8	60	0.125	289	330	1.18	314	410	1.64
9	60	0.16	393	354	2.23	405	440	2.43

**Table 4:** Differences in geometry and physical mechanisms for both types of tools.

Parameter	CBN Tool	Ceramic Tool
Dominant deformation	elastic-plastic	thermal plasticization
Turning marks	smeared	regular
Surface roughness	lower	higher
Surface character	physical-adhesive	geometric

**Table 5:** Key differences observed in microscopic images for CBN and ceramic tools.

Parameter	CBN Tool	Ceramic Tool
Surface Characteristics	blurred, non homogeneous	regular, periodic
Material Smearing	locally visible	sporadic
Turning marks	partially erased	clearly visible
Material Adhesion	strong	moderate
Representation of turning kinematics	partially disturbed	well preserved
Dominant Mechanism	elastic-plastic deformation and adhesion	material shearing
Topographic Character	physical adhesive	geometric

**Table 6:** Model regression statistics.

Parametr	Value
Multiple (R)	0.96
R squared (R^2^)	0.91
Adjusted R^2^	0.91
Standard error	0.174
Number of Observations	54

**Table 7:** Analysis of Variance (ANOVA).

	d.f.	SS	MS	F	p-Value
Regression	4.	15.6745	3.9186	128.9474	0.0000
Residual	48.	1.4587	0.0304		
Total	52.	17.1332			

## Data Availability

The original contributions presented in this study are included in the article. Further inquiries can be directed to the corresponding author.

## References

[1] Biffi C.A., Fiocchi J., Tuissi A. (2022). Relevant aspects of laser cutting of NiTi shape memory alloys. J. Mater. Res. Technol..

[2] Pelton A.R., Launey M.E., LePage W.S., Mitchell M.R., Ulmer J. (2024). Crack Formation and Pathways in Nitinol Biomedical Devices. Procedia Struct. Integr..

[3] Lopes J.d.S., Câmara P.B., Davis R., de Araujo C.J., da Silva M.B., Machado Á.R. (2024). Insights into the fabrication of microchannels on biomedical NiTinol. CIRP J. Manuf. Sci. Technol..

[4] DesRoches R., Delemont M. (2002). Seismic retrofit of simply supported bridges using shape memory alloys. Eng. Struct..

[5] Kowalczyk M. (2024). Analysis of Cutting Forces and Geometric Surface Structures in the Milling of NiTi Alloy. Materials.

[6] Wang G., Xia H., Huang W., Yang J., Liu B., Yuan L. (2022). Influence of Milling–Electrochemical Polishing on Corrosion Resistance of NiTi Shape Memory Alloy. Micromachines.

[7] Carbonaro D., Villa E., Gallo D., Morbiducci U., Audenino A.L., Chiastra C. (2024). Designing the mechanical behavior of NiTi self-expandable vascular stents by tuning the heat treatment parameters. J. Mech. Behav. Biomed. Mater..

[8] Obeidi M.A., Monu M., Hughes C., Bourke D., Dogu M.N., Francis J., Zhang M., Ahad I.U., Brabazon D. (2021). Laser beam powder bed fusion of nitinol shape memory alloy (SMA). J. Mater. Res. Technol..

[9] Sullivan S.J.L., Madamba D., Sivan S., Miyashiro K., Dreher M.L., Trépanier C., Nagaraja S. (2017). The effects of surface processing on in-vivo corrosion of Nitinol stents in a porcine model. Acta Biomater..

[10] Bhardwaj A., Ojha M., Garudapalli A., Gupta A.K. (2021). Microstructural, mechanical and strain hardening behaviour of NiTi alloy subjected to constrained groove pressing and ageing treatment. J. Mater. Process. Technol..

[11] Li X., Yang Y., Shen H., Zhou M., Huang B., Cui L., Hao S. (2025). Research progress on surface modification and coating technologies of biomedical NiTi alloys. Colloids Surf. B Biointerfaces.

[12] Kumar N., Nandan G., Chand S., Kumar A., Giri J., Elsharkawy E.R. (2025). A comprehensive review on non-conventional machining of Nickel-Titanium alloys: Techniques and surface integrity. Results Eng..

[13] Kowalczyk M., Tomczyk K. (2024). Quality of Machined Surface and Cutting Force When Milling NiTi Alloys. Materials.

[14] Zhao Y., Sun J. (2023). Study on the characteristics of phase in turning NiTi shape memory alloy. J. Manuf. Process..

[15] Wei J., Yang L., Wang G., Gong C., Yang F. (2024). Research status of cutting machining NiTi shape memory alloys: A comprehensive review. Front. Mater..

[16] Uddin H.M.W., Progoti Q.R.M., Mondal M.C., Arman S.E., Malitha S.B. (2025). Adaptive nickel–titanium shape memory alloy for smart systems: Mechanisms, manufacturing, and applications across biomedical, aerospace, civil, and energy. Mater. Today Commun..

[17] Dzogbewu T.C., de Beer D.J. (2024). Additive manufacturing of NiTi shape memory alloy and its industrial applications. Heliyon.

[18] Bertolini R., Bruschi S., Ghiotti A., Savio E., Ceseracciu L., Jawahir I.S. (2023). Surface integrity and superelastic response of additively manufactured Nitinol after heat treatment and finish machining. CIRP Ann.-Manuf. Technol..

[19] Mwangi J.W., Nguyen L.T., Bui V.D., Berger T., Zeidler H., Schubert A. (2019). Nitinol manufacturing and micromachining: A review of processes and their suitability in processing medical-grade nitinol. J. Manuf. Process..

[20] García V.D.-F., Vázquez M.I.P., Tejedor B., Suárez A., Freire Y. (2024). Comparative study of torsional and bending stress in NiTi, graphene, and GUM metal endodontic files by finite element analysis. Comput. Biol. Med..

[21] Zhao Y.-Z., Guo K., Sivalingam V., Li J.-F., Sun Q.-D., Zhu Z.-J., Sun J. (2021). Surface integrity evolution of machined NiTi shape memory alloys after turning process. Adv. Manuf..

[22] Bisaria H., Shandilya P. (2020). Surface integrity aspects for NiTi shape memory alloys during wire electric discharge machining: A review. J. Mater. Res..

[23] Asadi F., Martin U., Esmacher O., Crow R., Maheen M.H., Paredes M., Castaneda H. (2026). Corrosion Behavior of Nitinol Shape Memory Alloy Across Martensitic, Mixed, and Austenitic Phases. Metals.

[24] Wang X., Li Z., Wang Y., Wang Z., Chen Z., Liu J., Wang J., Wang G. (2026). The Effect of Micro-Cutting on the Residual Height of Surface Topography in NiTi Shape Memory Alloy Using a Small-Diameter Cutter. Coatings.

[25] Khalil A.N.M., Azmi A.I., Murad M.N., Ali M.A.M. (2018). The effect of cutting parameters on cutting force and tool wear in machining Nickel Titanium Shape Memory Alloy ASTM F2063 under Minimum Quantity Nanolubricant. Procedia CIRP.

[26] Marchenko E.S., Kozulin A.A., Yasenchuk Y.F., Vetrova A.V., Volinsky A.A., Zhang Y. (2022). Numerical and experimental study of porous NiTi anisotropy under compression. J. Mater. Res. Technol..

[27] Weinert K., Petzoldt V. (2004). Machining of NiTi based shape memory alloys. Mater. Sci. Eng. A.

[28] Liang Y., Yue L. (2022). Evolution and development: Engine-driven endodontic rotary nickel-titanium instruments. Int. J. Oral Sci..

[29] Kowalczyk M. (2017). Cutting forces during precise turning of NiTi shape memory alloy. Czas. Tech..

[30] Bertolini R., Tucci F., Ghiotti A., Jawahir I.S., Bruschi S. (2025). Analysis of tool wear and surface integrity in turning wrought and additively manufactured Ni_50.8_Ti_49.2_ shape memory alloys. Wear.

[31] Zhao Y., Li J., Guo K., Sivalingam V., Sun J. (2020). Study on chip formation characteristics in turning NiTi shape memory alloys. J. Manuf. Process..

[32] Vora J., Khanna S., Chaudhari R., Patel V.K., Paneliya S., Pimenov D.Y., Giasin K., Prakash C. (2022). Machining parameter optimization and experimental investigations of nano-graphene mixed electrical discharge machining of nitinol shape memory alloy. J. Mater. Res. Technol..

[33] Bai T., Wang C., Yu G., Kolmanovskyi M., Saelzer J., Kizaki T., Biermann D., Fang Z. (2025). Towards understanding the surface strengthening mechanism in negative rake angle cutting of additively manufactured stainless steel. CIRP Ann.-Manuf. Technol..

[34] (1997). Geometrical Product Specifications (GPS)—Surface Texture: Profile Method—Terms, Definitions and Surface Texture Parameters.

